# Supplementary material for: Reduced Neuroinflammation and Improved Functional Recovery after Traumatic Brain Injury by Prophylactic Diet Supplementation in Mice
Source: Nutrients. 2019 Jan 31;11(2):299. doi: 10.3390/nu11020299 (PMC6412510; doi:10.3390/nu11020299)
Supplement: Supplementary file 1 [file nutrients-11-00299-s001.pdf]

Table 1. Composition of GrandFusion Supplements

Blend #1: Fruit and Vegetable Blend (NF-216)

6 Essential Vitamins	Minimum Premix Claim Per 225.00 mg	
**Nutrient**	**% dv**	**Label Claim**
Vitamin A	50.00	2,500.000 IU
Vitamin C	50.00	30.000 mg
Vitamin D	50.00	200.000 IU
Vitamin E	50.00	15.000 IU
Vitamin B1	50.00	0.7500 mg
Vitamin B6	50.00	1.000 mg

Vegetable: Pwd Tomato, Broccoli, Carrot, Shitake Mushrooms
Fruit: Pwd Cranberry, Apple, Orange Made from 100% organic materials

Blend #2: Fruit Blend (NF-316)

6 Essential Vitamins	Minimum Premix Claim Per 225.00 mg	
**Nutrient**	**% dv**	**Label Claim**
Vitamin A	50.00	2,500.000 IU
Vitamin C	50.00	30.000 mg
Vitamin D	50.00	200.000 IU
Vitamin E	50.00	15.000 IU
Vitamin B1	50.00	0.7500 mg
Vitamin B6	50.00	1.000 mg

Fruit: Pwd Orange, Cranberry, Apple, Cherry, Blueberry, Strawberry, Shitake Mushrooms
Made from 100% organic materials

Blend #3: Vegetable Blend (NF-416)

6 Essential Vitamins	Minimum Premix Claim Per 225.00 mg	
**Nutrient**	**% dv**	**Label Claim**
Vitamin A	50.00	2,500.000 IU
Vitamin C	50.00	30.000 mg
Vitamin D	50.00	200.000 IU
Vitamin E	50.00	15.000 IU
Vitamin B1	50.00	0.7500 mg
Vitamin B6	50.00	1.000 mg

Vegetable: Pwd Spinach, Broccoli, Carrot, Tomato, Beet, Shitake Mushrooms
Made from 100% organic materials
